# Allyl­ammonium hydrogen oxalate hemihydrate

**DOI:** 10.1107/S1600536814015190

**Published:** 2014-07-05

**Authors:** Błażej Dziuk, Bartosz Zarychta, Krzysztof Ejsmont

**Affiliations:** a Faculty of Chemistry, University of Opole, Oleska 48, 45-052 Opole, Poland

**Keywords:** crystal structure

## Abstract

In the title hydrated mol­ecular salt, C_3_H_8_N^+^·C_2_HO_4_^−^·0.5H_2_O, the water O atom lies on a crystallographic twofold axis. The C=C—C—N torsion angle in the cation is 2.8 (3)° and the dihedral angle between the CO_2_ and CO_2_H planes in the anion is 1.0 (4)°. In the crystal, the hydrogen oxalate ions are linked by O—H⋯O hydrogen bonds, generating [010] chains. The allyl­ammonium cations bond to the chains through N—H⋯O and N—H⋯(O,O) hydrogen bonds. The water mol­ecule accepts two N—H⋯O hydrogen bonds and makes two O—H⋯O hydrogen bonds. Together, the hydrogen bonds generate (100) sheets.

## Related literature   

For the crystal structures of oxalic acid salts with aliphatic amines, see: Ejsmont (2006 ▶), (2007 ▶); Ejsmont & Zaleski (2006*a* ▶,*b* ▶); Vaidhyanathan *et al.* (2001 ▶, 2002 ▶); MacDonald *et al.* (2001 ▶) For information on the Cambridge Database, see: Allen (2002 ▶).
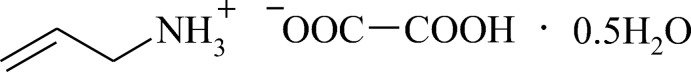


## Experimental   

### 

#### Crystal data  

C_3_H_8_N^+^·C_2_HO_4_^−^·0.5H_2_O
*M* *_r_* = 156.14
Monoclinic, 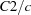

*a* = 21.578 (3) Å
*b* = 5.6521 (4) Å
*c* = 13.8629 (17) Å
β = 118.415 (17)°
*V* = 1487.0 (3) Å^3^
*Z* = 8
Mo *K*α radiation
μ = 0.12 mm^−1^
*T* = 100 K
0.33 × 0.18 × 0.14 mm

#### Data collection  

Oxford Diffraction Xcalibur diffractometer
4525 measured reflections
1376 independent reflections
958 reflections with *I* > 2σ(*I*)
*R*_int_ = 0.045

#### Refinement  

*R*[*F* ^2^ > 2σ(*F* ^2^)] = 0.043
*wR*(*F* ^2^) = 0.098
*S* = 0.90
1376 reflections
136 parameters
All H-atom parameters refined
Δρ_max_ = 0.26 e Å^−3^
Δρ_min_ = −0.27 e Å^−3^

### 

Data collection: *CrysAlis CCD* (Oxford Diffraction, 2008 ▶); cell refinement: *CrysAlis RED* (Oxford Diffraction, 2008 ▶); data reduction: *CrysAlis RED* ; program(s) used to solve structure: *SHELXS97* (Sheldrick, 2008 ▶); program(s) used to refine structure: *SHELXL97* (Sheldrick, 2008 ▶); molecular graphics: *SHELXTL* (Sheldrick, 2008 ▶); software used to prepare material for publication: *SHELXL97*.

## Supplementary Material

Crystal structure: contains datablock(s) global, I. DOI: 10.1107/S1600536814015190/hb7243sup1.cif

Structure factors: contains datablock(s) I. DOI: 10.1107/S1600536814015190/hb7243Isup2.hkl

Supporting information file. DOI: 10.1107/S1600536814015190/hb7243Isup3.cml

CCDC reference: 1010915

Additional supporting information:  crystallographic information; 3D view; checkCIF report

## Figures and Tables

**Table 1:** Hydrogen-bond geometry (Å, °)

*D*—H⋯*A*	*D*—H	H⋯*A*	*D*⋯*A*	*D*—H⋯*A*
N1—H1*B*⋯O9	0.98 (3)	1.86 (3)	2.825 (2)	169 (2)
N1—H1*A*⋯O11	0.98 (3)	1.82 (3)	2.769 (2)	161 (2)
N1—H1*C*⋯O8^i^	0.91 (3)	2.19 (3)	3.014 (2)	151 (2)
N1—H1*C*⋯O10^i^	0.91 (3)	2.16 (3)	2.853 (2)	132 (2)
O7—H7⋯O10^ii^	0.94 (3)	1.62 (3)	2.5563 (19)	179 (4)
O11—H11⋯O9^iii^	0.88 (3)	1.86 (3)	2.739 (2)	176 (3)

Symmetry codes: (i) 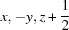; (ii) 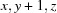; (iii) 
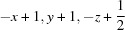
.

**Fig. 1.**
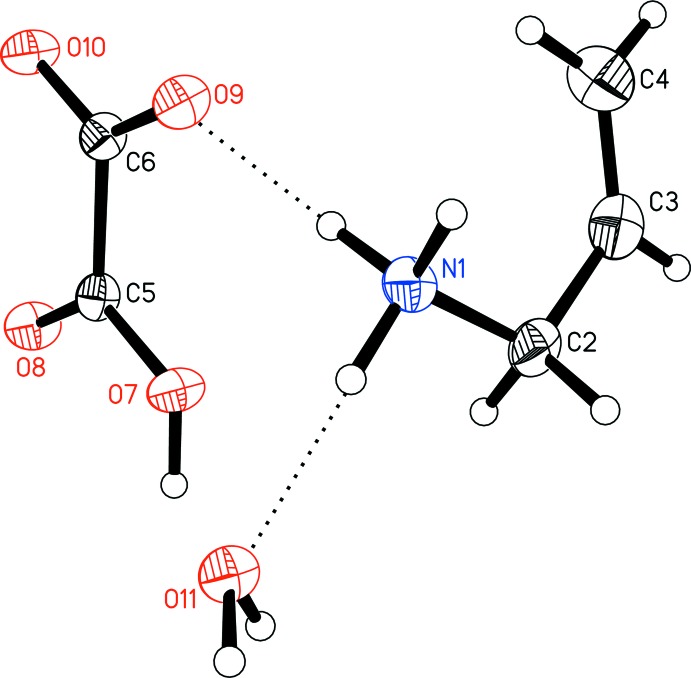
The molecular structure of (I), showing 50% displacement ellipsoids (arbitrary spheres for the H atoms). Hydrogen bonds are shown as dotted lines.

**Fig. 2.**
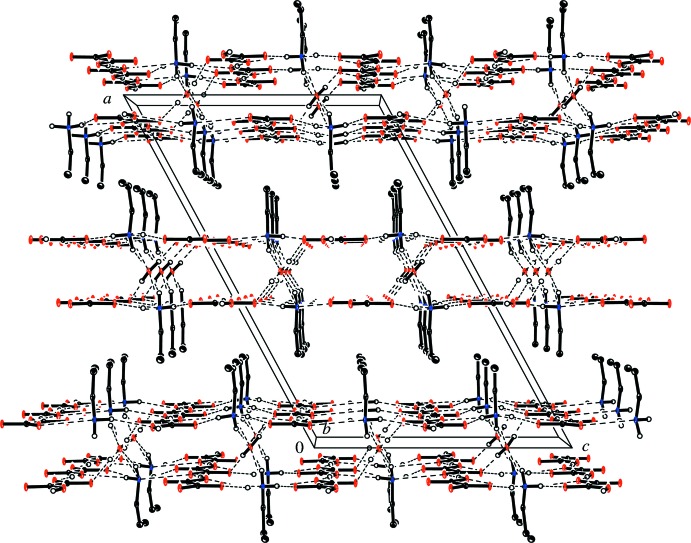
The packing diagram of (I), viewed along the b axis, showing the intermolecular hydrogen-bonding scheme (dashed lines).

## supplementary crystallographic information

### S1. Comment

Oxalic acid, together with its anions, is one of the best building blocks for the construction of supramolecular structures based on hydrogen bonds. The adducts of oxalic acid and aliphatic amines have been examined by single-crystal X-ray diffraction and other techniques. Three types of characteristic structural motifs are present: (i) linear chains of dicarboxylic acids formed by strong hydrogen bonds; (ii) dimers of dicarboxylic acid molecules; (iii) isolated oxalate monoanions or dianion units (MacDonald *et al.*, 2001; Vaidhyanathan *et al.*, 2001, 2002); Ejsmont, 2006, 2007; Ejsmont & Zaleski 2006*a*, 2006*b*).

The crystal structure of the title salt, (I), consists of allyloammonium cations, hydrogen oxalate anions and water molecules (Fig. 1). A search of the Cambridge Structural Database (CSD; CONQUEST Version 1.16; Allen, 2002) afforded that the geometrical parameters of the allyloammonium cation (Table 1) compare well with those found in other crystal structures which include this cation (Allen, 2002). The oxalate monoanions are nearly planar and are connected to each other by strong O—H···O hydrogen bonds along the *b* axis. The allyloammonium cations form N—H···O H atoms bonds to the anions and water molecules (Fig. 2 and Table 2).

### S2. Experimental

Colourless prisms of (I) were grown at room temperature by slow evaporation of an aqueous solution of allylamine and oxalic acid in a 1:1 stoichiometric ratio.

### S3. Refinement

All H atoms were positioned geometrically and their parameters are refined independently.

### Crystal data

**Table:**

C_3_H_8_N^+^·C_2_HO_4_^−^·0.5H_2_O	*F*(000) = 664
*M**_r_* = 156.14	*D*_x_ = 1.395 Mg m^−^^3^
Monoclinic, *C*2/*c*	Mo *K*α radiation, λ = 0.71073 Å
Hall symbol: -C 2yc	Cell parameters from 4525 reflections
*a* = 21.578 (3) Å	θ = 3.3–25.5°
*b* = 5.6521 (4) Å	µ = 0.12 mm^−^^1^
*c* = 13.8629 (17) Å	*T* = 100 K
β = 118.415 (17)°	Prism, colourless
*V* = 1487.0 (3) Å^3^	0.33 × 0.18 × 0.14 mm
*Z* = 8	

### Data collection

**Table:**

Oxford Diffraction Xcalibur diffractometer	958 reflections with *I* > 2σ(*I*)
Radiation source: fine-focus sealed tube	*R*_int_ = 0.045
Graphite monochromator	θ_max_ = 25.5°, θ_min_ = 3.3°
Detector resolution: 1024 x 1024 with blocks 2 x 2 pixels mm^-1^	*h* = −26→26
ω–scan	*k* = −6→5
4525 measured reflections	*l* = −16→16
1376 independent reflections	

### Refinement

**Table:**

Refinement on *F*^2^	Primary atom site location: structure-invariant direct methods
Least-squares matrix: full	Secondary atom site location: difference Fourier map
*R*[*F*^2^ > 2σ(*F*^2^)] = 0.043	Hydrogen site location: inferred from neighbouring sites
*wR*(*F*^2^) = 0.098	All H-atom parameters refined
*S* = 0.90	*w* = 1/[σ^2^(*F*_o_^2^) + (0.0628*P*)^2^] where *P* = (*F*_o_^2^ + 2*F*_c_^2^)/3
1376 reflections	(Δ/σ)_max_ < 0.001
136 parameters	Δρ_max_ = 0.26 e Å^−^^3^
0 restraints	Δρ_min_ = −0.27 e Å^−^^3^

### Special details

**Table:**

Geometry. All e.s.d.'s (except the e.s.d. in the dihedral angle between two l.s. planes) are estimated using the full covariance matrix. The cell e.s.d.'s are taken into account individually in the estimation of e.s.d.'s in distances, angles and torsion angles; correlations between e.s.d.'s in cell parameters are only used when they are defined by crystal symmetry. An approximate (isotropic) treatment of cell e.s.d.'s is used for estimating e.s.d.'s involving l.s. planes.
Refinement. Refinement of *F*^2^ against ALL reflections. The weighted *R*-factor *wR* and goodness of fit *S* are based on *F*^2^, conventional *R*-factors *R* are based on *F*, with *F* set to zero for negative *F*^2^. The threshold expression of *F*^2^ > σ(*F*^2^) is used only for calculating *R*-factors(gt) *etc*. and is not relevant to the choice of reflections for refinement. *R*-factors based on *F*^2^ are statistically about twice as large as those based on *F*, and *R*- factors based on ALL data will be even larger.

### Fractional atomic coordinates and isotropic or equivalent isotropic displacement parameters (Å^2^)

**Table:**

	*x*	*y*	*z*	*U*_iso_*/*U*_eq_	
N1	0.40092 (10)	0.2394 (3)	0.23240 (15)	0.0196 (4)	
H1A	0.4411 (13)	0.348 (4)	0.2556 (19)	0.033 (6)*	
H1B	0.4018 (14)	0.132 (5)	0.177 (2)	0.050 (8)*	
H1C	0.4052 (14)	0.154 (5)	0.291 (2)	0.046 (8)*	
C2	0.33560 (12)	0.3802 (4)	0.18227 (19)	0.0231 (5)	
H2A	0.3367 (11)	0.466 (4)	0.1271 (18)	0.021 (5)*	
H2B	0.3376 (12)	0.486 (4)	0.233 (2)	0.032 (6)*	
C3	0.27053 (13)	0.2364 (4)	0.13940 (19)	0.0284 (5)	
H3	0.2311 (12)	0.325 (4)	0.1035 (18)	0.026 (6)*	
C4	0.26561 (15)	0.0080 (5)	0.1452 (2)	0.0327 (6)	
H4A	0.2203 (14)	−0.075 (4)	0.111 (2)	0.042 (7)*	
H4B	0.3053 (13)	−0.085 (4)	0.1769 (19)	0.028 (6)*	
C5	0.41682 (10)	0.1591 (3)	−0.03835 (16)	0.0167 (5)	
C6	0.41572 (10)	−0.0892 (3)	0.00740 (16)	0.0177 (5)	
O7	0.41788 (8)	0.3311 (2)	0.02573 (11)	0.0211 (4)	
H7	0.4173 (16)	0.482 (6)	−0.002 (3)	0.077 (10)*	
O8	0.41599 (8)	0.1838 (2)	−0.12581 (11)	0.0228 (4)	
O9	0.41549 (8)	−0.1043 (2)	0.09658 (11)	0.0231 (4)	
O10	0.41539 (8)	−0.2570 (2)	−0.05214 (11)	0.0221 (4)	
O11	0.5000	0.5720 (4)	0.2500	0.0199 (5)	
H11	0.5269 (14)	0.672 (5)	0.302 (2)	0.055 (9)*	

### Atomic displacement parameters (Å^2^)

**Table:**

	*U*^11^	*U*^22^	*U*^33^	*U*^12^	*U*^13^	*U*^23^
N1	0.0289 (11)	0.0222 (10)	0.0119 (8)	−0.0017 (8)	0.0133 (8)	0.0000 (8)
C2	0.0356 (13)	0.0234 (12)	0.0166 (11)	0.0039 (10)	0.0175 (10)	0.0007 (10)
C3	0.0273 (14)	0.0331 (14)	0.0255 (12)	0.0051 (11)	0.0132 (11)	0.0020 (10)
C4	0.0290 (14)	0.0362 (15)	0.0294 (13)	−0.0024 (12)	0.0110 (11)	0.0021 (12)
C5	0.0189 (11)	0.0201 (10)	0.0113 (10)	−0.0001 (8)	0.0073 (8)	−0.0027 (8)
C6	0.0202 (11)	0.0194 (10)	0.0136 (10)	0.0009 (8)	0.0082 (9)	−0.0002 (8)
O7	0.0383 (9)	0.0158 (7)	0.0148 (7)	−0.0003 (7)	0.0172 (7)	0.0003 (6)
O8	0.0394 (9)	0.0224 (8)	0.0128 (7)	−0.0011 (6)	0.0173 (7)	0.0005 (6)
O9	0.0416 (9)	0.0222 (8)	0.0145 (8)	0.0049 (6)	0.0206 (7)	0.0037 (6)
O10	0.0395 (9)	0.0172 (7)	0.0148 (7)	−0.0003 (6)	0.0170 (7)	−0.0014 (6)
O11	0.0283 (12)	0.0207 (11)	0.0119 (10)	0.000	0.0107 (9)	0.000

### Geometric parameters (Å, º)

**Table:**

N1—C2	1.473 (3)	C4—H4A	0.98 (3)
N1—H1A	0.98 (3)	C4—H4B	0.92 (2)
N1—H1B	0.98 (3)	C5—O8	1.212 (2)
N1—H1C	0.91 (3)	C5—O7	1.309 (2)
C2—C3	1.480 (3)	C5—C6	1.545 (3)
C2—H2A	0.92 (2)	C6—O9	1.242 (2)
C2—H2B	0.91 (2)	C6—O10	1.255 (2)
C3—C4	1.301 (3)	O7—H7	0.94 (3)
C3—H3	0.91 (2)	O11—H11	0.88 (3)
			
C2—N1—H1A	108.3 (13)	C4—C3—H3	120.1 (14)
C2—N1—H1B	109.5 (15)	C2—C3—H3	112.3 (14)
H1A—N1—H1B	108 (2)	C3—C4—H4A	122.4 (14)
C2—N1—H1C	112.2 (17)	C3—C4—H4B	120.8 (14)
H1A—N1—H1C	110 (2)	H4A—C4—H4B	116.6 (19)
H1B—N1—H1C	109 (2)	O8—C5—O7	125.49 (18)
N1—C2—C3	113.87 (18)	O8—C5—C6	121.27 (17)
N1—C2—H2A	106.4 (13)	O7—C5—C6	113.24 (15)
C3—C2—H2A	110.6 (13)	O9—C6—O10	126.98 (18)
N1—C2—H2B	108.0 (14)	O9—C6—C5	118.63 (16)
C3—C2—H2B	111.1 (15)	O10—C6—C5	114.39 (16)
H2A—C2—H2B	106.5 (19)	C5—O7—H7	113.9 (19)
C4—C3—C2	127.6 (2)		
			
N1—C2—C3—C4	2.8 (3)	O8—C5—C6—O10	1.4 (3)
O8—C5—C6—O9	−178.8 (2)	O7—C5—C6—O10	−179.34 (17)
O7—C5—C6—O9	0.4 (3)		

### Hydrogen-bond geometry (Å, º)

**Table:**

*D*—H···*A*	*D*—H	H···*A*	*D*···*A*	*D*—H···*A*
N1—H1*B*···O9	0.98 (3)	1.86 (3)	2.825 (2)	169 (2)
N1—H1*A*···O11	0.98 (3)	1.82 (3)	2.769 (2)	161 (2)
N1—H1*C*···O8^i^	0.91 (3)	2.19 (3)	3.014 (2)	151 (2)
N1—H1*C*···O10^i^	0.91 (3)	2.16 (3)	2.853 (2)	132 (2)
O7—H7···O10^ii^	0.94 (3)	1.62 (3)	2.5563 (19)	179 (4)
O11—H11···O9^iii^	0.88 (3)	1.86 (3)	2.739 (2)	176 (3)

Symmetry codes: (i) *x*, −*y*, *z*+1/2; (ii) *x*, *y*+1, *z*; (iii) −*x*+1, *y*+1, −*z*+1/2.
